# Supplementary material for: MPicker: visualizing and picking membrane proteins for cryo-electron tomography
Source: Nat Commun. 2025 Jan 8;16:472. doi: 10.1038/s41467-024-55767-w (PMC11707294; doi:10.1038/s41467-024-55767-w)
Supplement: Supplementary file 1 — Supplementary Information [file 41467_2024_55767_MOESM1_ESM.pdf]

Supplementary Information for:

MPicker: Visualizing and Picking Membrane Proteins for Cryo-Electron Tomography

Authors: Xiaofeng Yan, Shudong Li, Weilin Huang, Hao Wang, Tianfang Zhao, Mingtao Huang, Niyun Zhou, Yuan Shen, Xueming Li

Inventory of Supporting Information:

Supplementary Table 1 2
Supplementary Figure 1 3
Supplementary Figure 2 4
Supplementary Figure 3 5
Supplementary Figure 4 6
Supplementary Figure 5 7
Supplementary Figure 6 8
Supplementary Figure 7 9
Supplementary Figure 8 10
Supplementary Figure 9 12
Supplementary Figure 10 13
Supplementary Figure 11 14

Supplementary Tables

Supplementary Table 1. Functional comparison of five software supporting particle picking of membrane proteins in cryoET tomogram. Five software packages, sharing similar functional objectives with MPicker either partially or entirely within the workflow, were selected for comparison. We listed and compared the essential functions for analyzing, visualizing, and picking membrane proteins.

Software name	**MPicker**	**Membranorama**	**Dynamo catalogue**	**PySeg**	**MemBrain**
Inputs	Membrane mask, or labeled points, or triangle mesh[a]	Triangle mesh	Labeled points	Membrane mask	Triangle mesh
Flattened tomogram generation	✓	×	×	×	×
Triangle mesh generation	✓	×	✓	×	×
Membrane segmentation	✓	×	×	✓[b]	×
Surface rendering in 3D with grey level	✓	✓	×	×	×
Normal vector generation	✓	✓	✓	✓	✓
Manual particle picking	In the flattened tomogram	On the 3D surface	In the raw tomogram	/[c]	/[c]
Manual in-plane angle estimation	✓	✓	×	×	×
Automated particle picking	✓[d]	×	×[e]	✓	✓
Automated in-plane angle estimation	✓[f]	×	×	×	✓

[a]: Flattening the triangle mesh relies on the software OptCuts for the parameterization.

[b]: This relies on the software TomoSegMemTV.

[c]: The software is designed for automated particle picking.

[d]: This relies on the software EPicker.

[e]: It supports oversampling particles on the surface.

[f]: This relies on the software THUNDER for the 2D classification.

Supplementary Figures

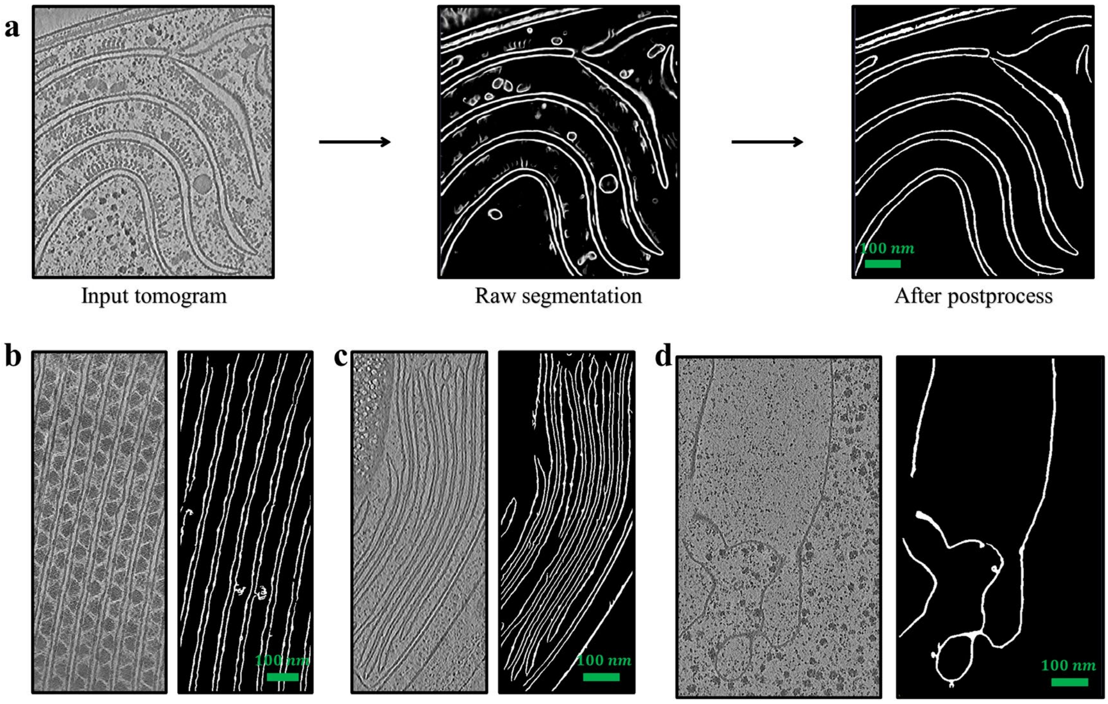

Supplementary Figure 1. Membrane segmentation in MPicker. a, Membrane segmentation process illustrated using a cyanobacterium tomogram (**Fig. 2b**). The pre-trained neural network calculated a continuous score (0 to 1) to create a raw segmentation map. This was followed by post-processing to finalize the membrane mask, distinguishing membranes with a score of 1 (white) from other regions with a score of 0 (black). **b,** Segmentation result of **Fig. 4a**. **c,** Segmentation result of **Fig. 4c**. **d,** Segmentation result of **Supplementary Fig. 3a**.

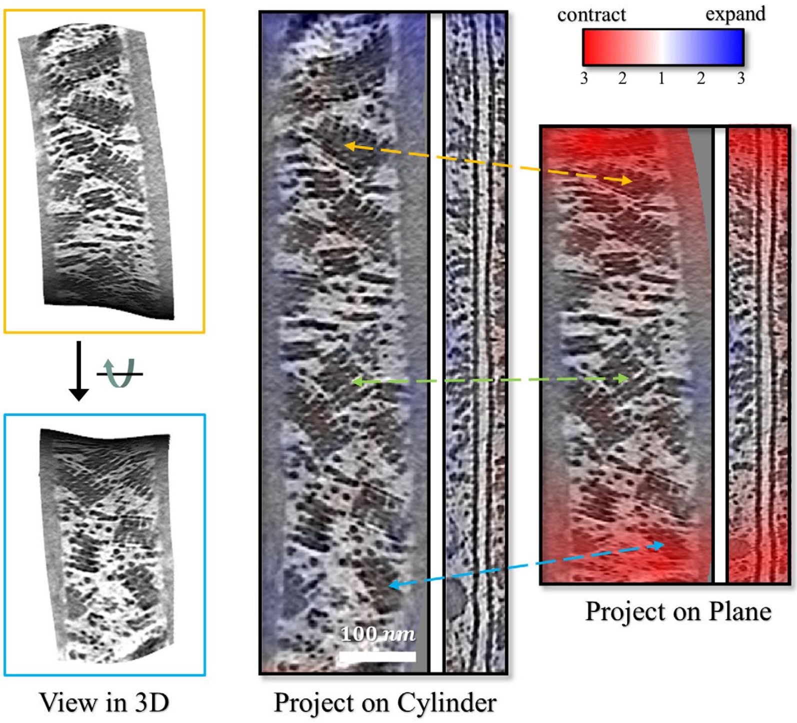

Supplementary Figure 2. Comparison of projections on a cylindrical surface or a plane. A curved surface (the same as **Fig. 2d**) was projected on a cylindrical surface and plane. The area distortions are presented using a color map. Taking the 3D view of the surface as a reference, the plane projection showed significant distortion at both ends, in contrast to the cylindrical projection. The corresponding locations in the two projections are indicated by dashed lines of different colors.

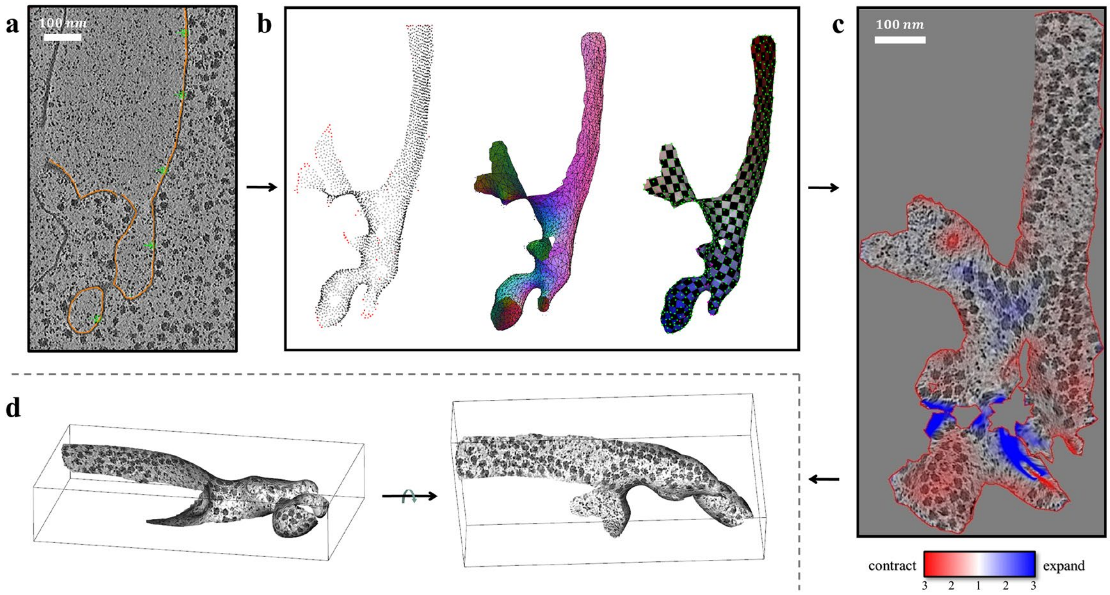

Supplementary Figure 3. Flattening a membrane represented in a triangle mesh. **a,** Representative view of the *Chlamydomonas* ER tomogram (EMD-10409). The starting points (orange) were extracted from the membrane mask (**Supplementary Fig. 1d**) based on the seed points (green). **b,** Procedure for generating a triangle mesh with texture coordinates (the result of parameterization) from starting points. The process begins with the generation of a downsampled point cloud from the starting points (left), where the outliers (red) are removed. Poisson surface reconstruction in Open3D was then carried out to obtain a triangle mesh (middle). Finally, OptCuts software was used for parameterization and to generate the texture coordinates (the checkerboard pattern shown in the right panel), which correspond to the coordinates in the central slice of the flattened tomogram. The color gradient from blue to red represents the y-coordinates of the flattened tomogram. The green dots on the surface represent the points used for thin-plate spline (TPS) interpolation during flattening. **c,** an *x*–*y* slice of the flattened tomogram. The area distortion is represented using a color map. **d,** 3D views of the *x*–*y* slice in **c**.

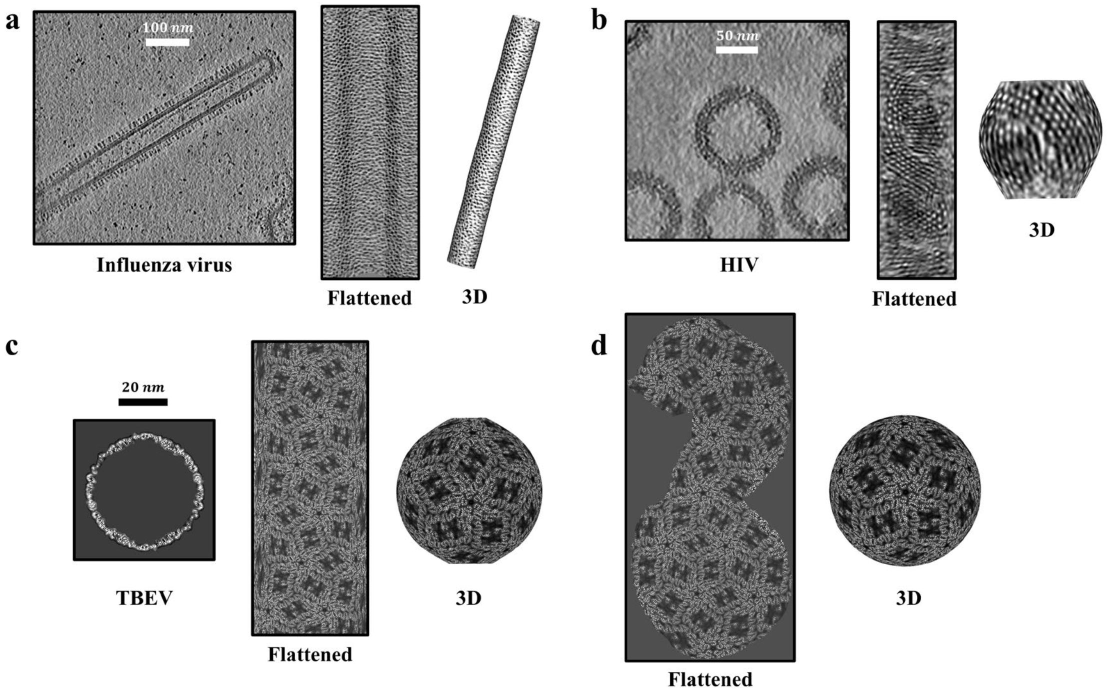

Supplementary Figure 4. Examples of the flattened cylindrical and spherical surface. To illustrate the performance of MPicker on flattening cylindrical and spherical surfaces, we chose three viruses as examples. **a,** Flattening the outer surface of an influenza virion in a tomogram (EMD-11075) by projecting it on a cylindrical surface. **b,** Flattening the outer surface of an HIV virus-like particle in a tomogram (TS_43 in EMPIAR-10164) by projecting it on a cylindrical surface. **c,** Flattening a density map (EMD-19003) of the TBEV virion by projecting it on a cylindrical surface. The membrane of TBEV was modeled as a sphere with the diameter of 46 nm. **d,** Flattening the same TBEV virion in **c** by triangle-mesh-based method. The processing workflow was the same as that used in **Supplementary Fig. 3**.

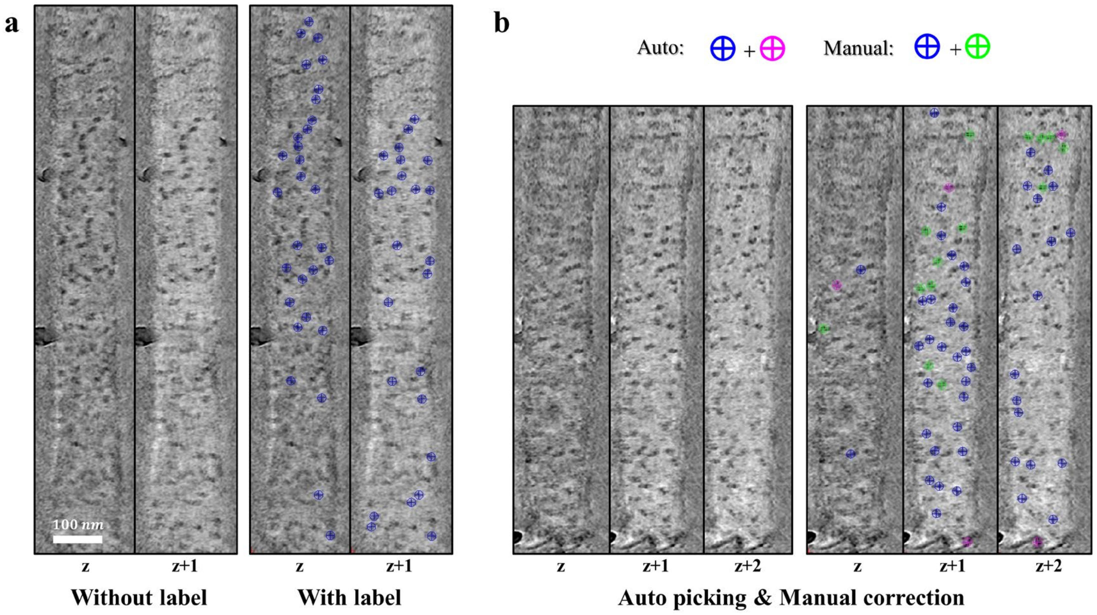

Supplementary Figure 5. Particle picking in flattened tomograms using EPicker. **a,** Example of particle labeling. Particles with clear features in the *x–y* slices of a thylakoid membrane flattened tomogram were labeled (blue). The same particle may be shown on two adjacent slices (z and $z + 1$), and hence labeled twice. **b,** Example of particle picking. Three adjacent *x–y* slices were selected for particle picking. Particles were automatically picked using EPicker (blue and magenta). Duplicate picking of the same particle shown on different slices was removed using MPicker. Subsequent manual screening removed incorrect particles (magenta) and added missing particles (green). **a** and **b** share the same scale bar.

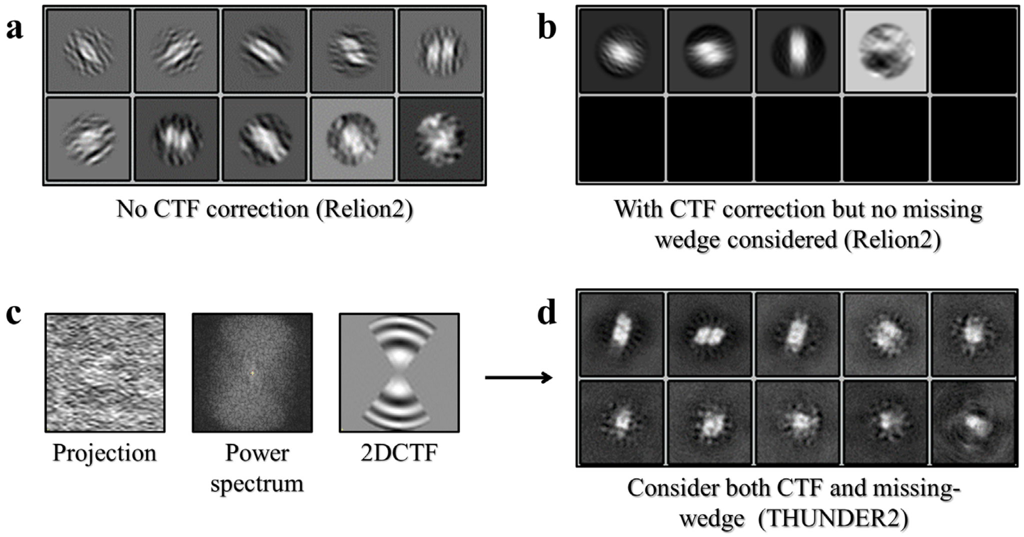

Supplementary Figure 6. 2D classification with different weighting algorithms. a, 2D classification without additional weighting. **b,** 2D classification with contrast transfer function (CTF) weighting only. **c,** Examples of particle projection, corresponding power spectrum, and 2DCTF. The power spectrum (plotted using IMOD) of the projection shows the influence of the missing wedge in the Fourier space, which is consistent with the 2DCTF sliced from the 3DCTF. **d,** 2D classification with the 2DCTF weighing (CTF + missing wedge). 2D class averages in **a**, **b**, and **d** are sorted in the descending order of occupancy.

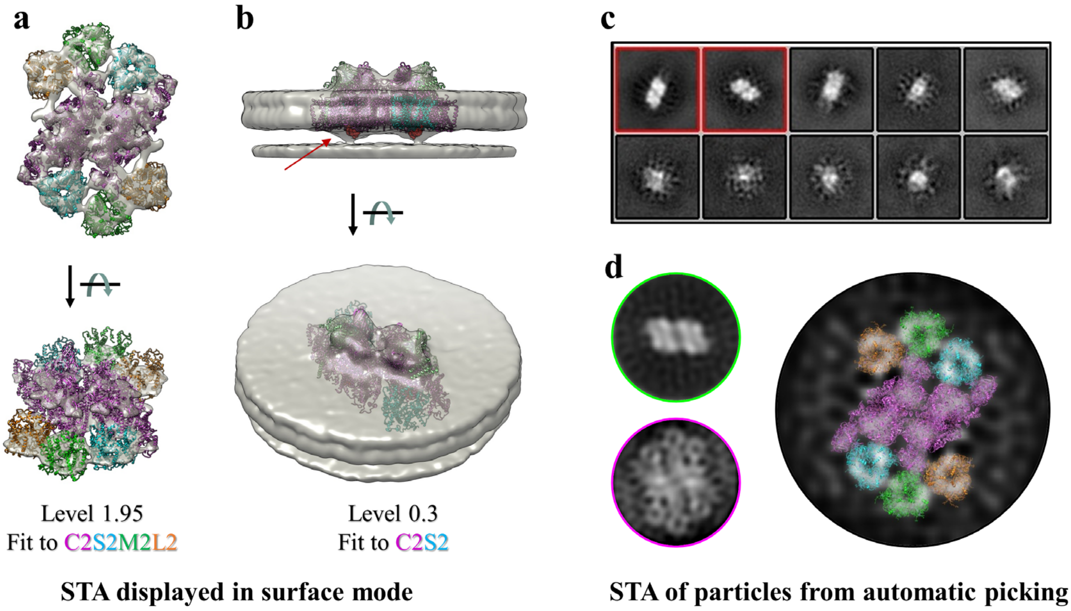

Supplementary Figure 7. STA of PSII. a, The final map of PSII–LHCII. A high contour level (1.95) was used to reveal the transmembrane region density. The PSII–LHCII supercomplex C2S2M2L2 model (PDB: 6KAD) was fitted to the map. Components C, S, M, and L are drawn in different colors, and the numbers following the letters (C, S, M, and L) indicate the number of components in the supercomplex. **b,** Visualization of the extramembrane region using a low contour level (0.3). The C2S2 model (PDB: 6KAC) was docked into the map. The difference in the C component (core) between the two models, C2S2M2L2 (PDB: 6KAD) and C2S2 (PDB: 6KAC), was that the C component in C2S2 model contained more subunits: PsbP (light green), PsbQ (light green), PsbR (light green), small luminal protein (light green), and unidentified stromal protein (USP) (red). The red arrow indicates the USP region. **c,** 2D classification of 5798 particles automatically picked by EPicker without manual correction. **d**, STA using 1863 particles in two selected 2D classes (labeled by red boxes in **c**). The same computational steps outlined in **Fig. 5c–f** were used here.

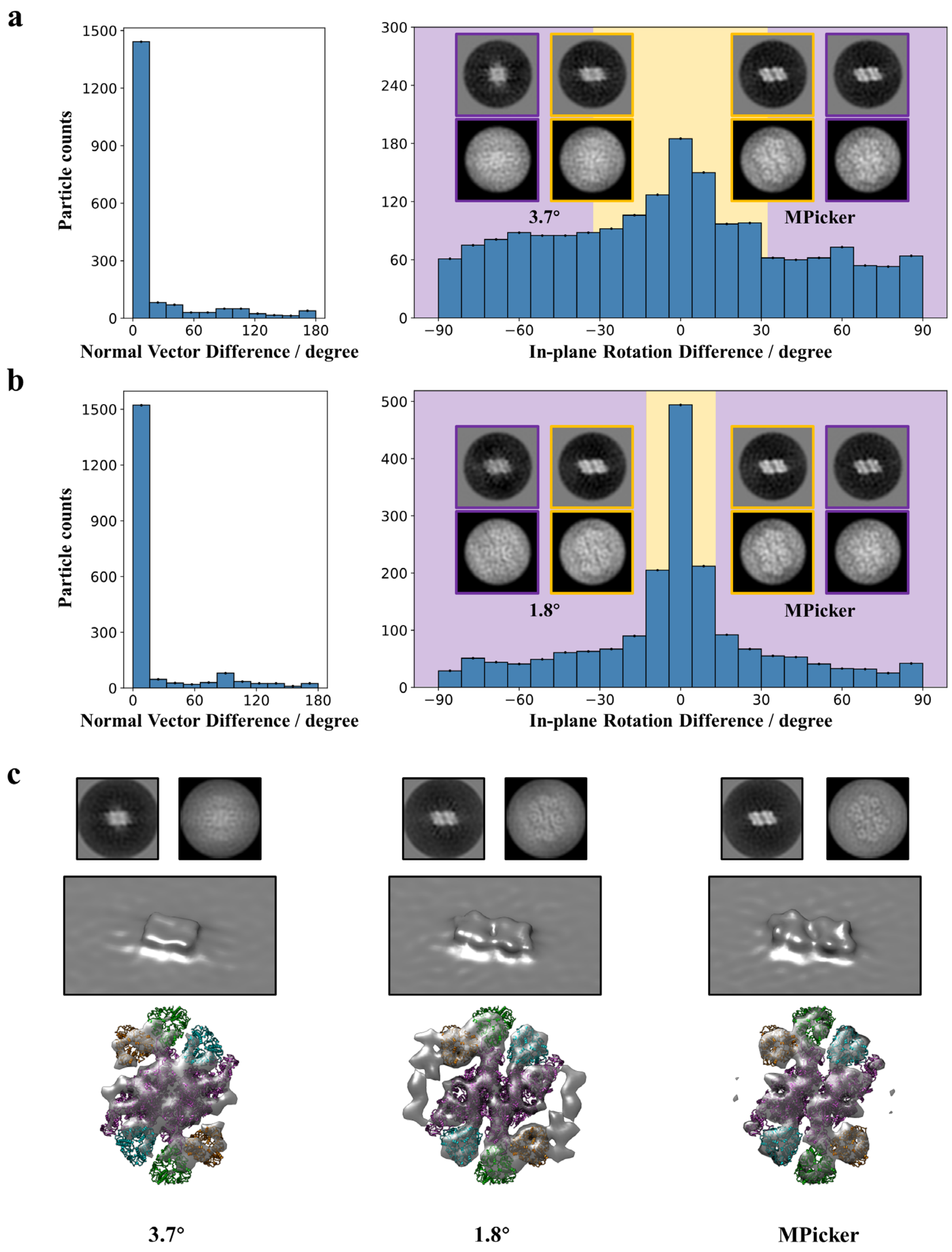

Supplementary Figure 8. STA results of PSII with different initial angular sampling. a, Histogram of the distribution of the angle differences between the STA result from scratch with the initial angular sampling step of 3.7° and that from MPicker (**Fig. 5e**). The left panel shows the distribution of the angular differences of the normal vectors of all particles. The right panel shows the distribution of the differences in the in-plane rotation angles of all particles. The inset figures show the section views of the reconstruction results, where the top and bottom images are the x–y slices crossing the extramembrane and transmembrane regions, respectively. The purple indicates the group where the particles had large in-plane angle differences, and the orange indicates the group where the particles had small differences. **b,** Histogram of the distribution of

the angle differences between the STA result from scratch with the initial angular sampling step of 1.8° and that from MPicker. A similar analysis was carried out as in **a**. **c,** Final density maps obtained by three different initial orientation settings. Two of them were conventional refinements without the initial orientation but with a global angular sampling step of 3.7° and 1.8° respectively. The other one used the coarse orientation estimated by MPicker as the initial orientation. The first row shows the x–y slices crossing the extramembrane and transmembrane regions. The second and third rows show the extramembrane and transmembrane regions using a low and high contour level.

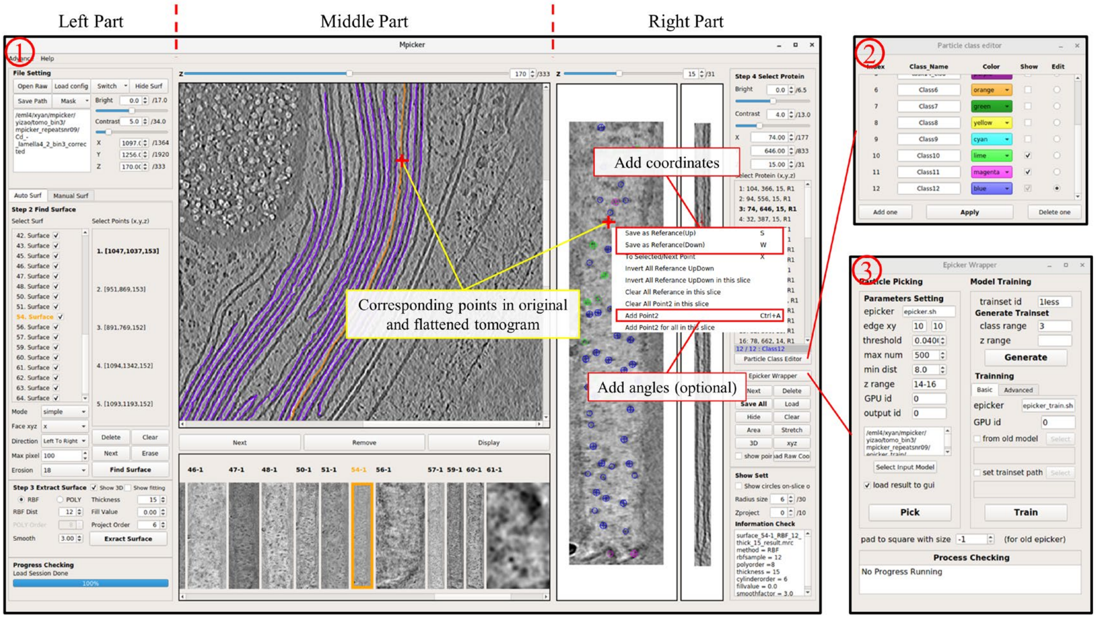

Supplementary Figure 9. The graphical user interface (GUI) of MPicker. The main GUI of MPicker (labeled 1) can be divided into three parts. The left part contains a list of the extracted surfaces and the parameters used for the input/output, surface extraction, and surface flattening. The middle part shows the raw tomogram and a list of flattened tomograms. The right part shows the flattened tomogram and contains tools for particle selection and visualization, such as an EPicker wrapper and a function to show distortions in color. MPicker allows users to extract and flatten multiple membranes. The actual position of a flattened tomogram point is displayed in a raw tomogram in real time (red crosses and yellow lines). The particle positions and orientations in the flattened tomograms can be manually labeled (red boxes). Users can independently pick various types of particles and display them in different colors, which can be controlled using a GUI labeled 2. The EPicker wrapper (labeled 3) allows the user to train the model and automatically pick particles.

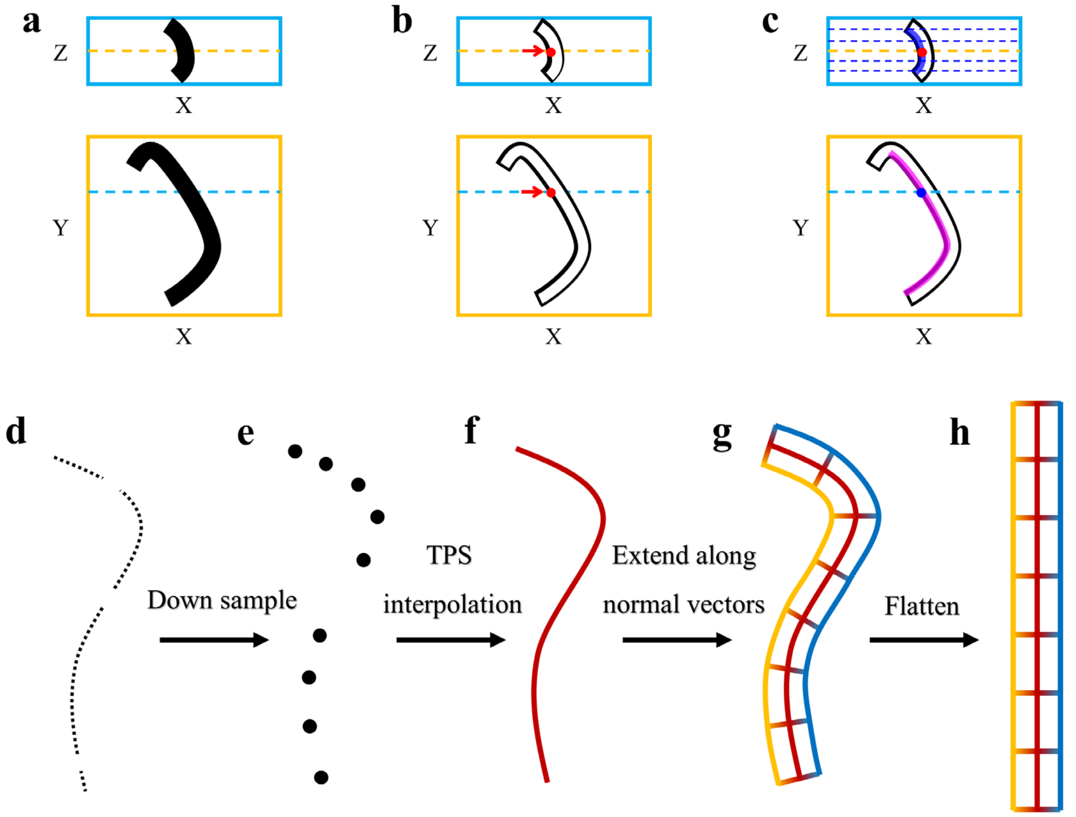

Supplementary Figure 10. Schematic diagram of the membrane flattening principle. a, A schematic diagram of membrane mask. Membranes are shown in black. The position of the *x*–*y* slice (orange) in the *x*–*z* slice (cyan) is indicated by an orange dashed line, and the position of the *x*–*z* slice in the *x*–*y* slice is indicated by a cyan dashed line. **b,** Boundary of the mask. Red points represent seed points. The horizontal red arrow indicates that a membrane approximately perpendicular to the *x*-axis is expected. **c,** Schematic of surface extraction based on the mask boundary. A curve (blue) in the *x*–*z* slice was obtained starting from the seed point (red). Then, in each *x*–*y* slice (marked by blue and orange dashed lines) passed by the curve, a curve (magenta) was obtained, starting from the intersection point (blue). Together, these curves form the starting points. In practice, all "curves" and "surfaces" mentioned here are composed of points (voxels). **d–h,** Schematic for obtaining a flattened tomogram from the starting points. Here, we assume that the surface is perfectly cylindrical so that it can be represented in 2D images.

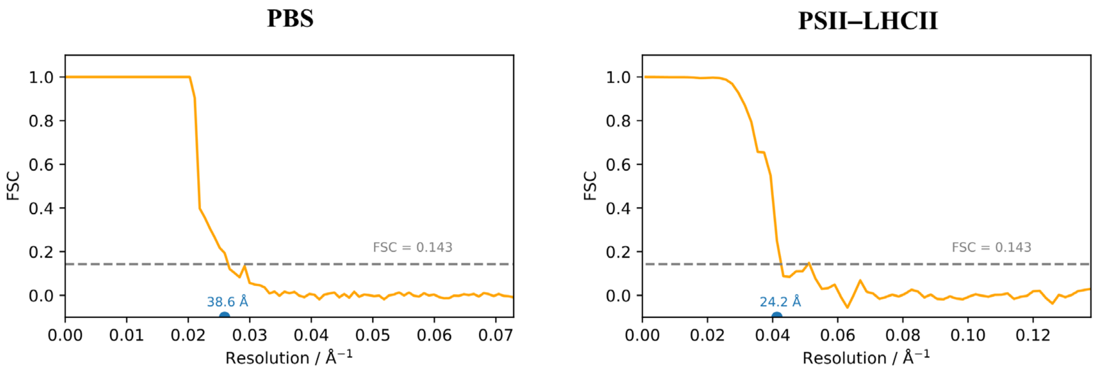

Supplementary Figure 11. FSC curves of 3D reconstruction of the PBS and PSII–LHCII complex.
