# Supplementary material for: MPicker: visualizing and picking membrane proteins for cryo-electron tomography
Source: Nat Commun. 2025 Jan 8;16:472. doi: 10.1038/s41467-024-55767-w (PMC11707294; doi:10.1038/s41467-024-55767-w)
Supplement: Supplementary file 2 — Description of Additional Supplementary Files [file 41467_2024_55767_MOESM2_ESM.pdf]

Description of Additional Supplementary Files

File Name: Supplementary Movie 1

Description: The movie of flattened tomogram and raw tomogram of the HeLa cell nuclear membrane shown in Figure 3. When the cursor (red point) goes through the flattened tomogram (left) from the bottom to the top, the corresponding position in the raw tomogram (right) moves as shown by the cursor. For the flattened tomogram, to enhance the contrast of the membrane, 61 y–z slices (a total of 103 nm thick) were summed to get the final slice, the same processing was done for displaying the x–z slices.
